# Automated volumetric radiomic analysis of breast cancer vascularization improves survival prediction in primary breast cancer

**DOI:** 10.1038/s41598-020-60393-9

**Published:** 2020-02-28

**Authors:** Matthias Dietzel, Rüdiger Schulz-Wendtland, Stephan Ellmann, Ramy Zoubi, Evelyn Wenkel, Matthias Hammon, Paola Clauser, Michael Uder, Ingo B. Runnebaum, Pascal A. T. Baltzer

**Affiliations:** 1 Department of Radiology, University Hospital Erlangen, Maximiliansplatz 3, 91054 Erlangen, Germany; 2 Department of Biomedical Imaging and Image-Guided Therapy, Division of Molecular and Gender Imaging, Medical University of Vienna, Waehringer-Guertel 18-20, Vienna, Austria; 3 Radiologische Gemeinschaftspraxis Ibbenbüren, Bergstraße 1, 49477 Ibbenbüren, Germany; 4 Department of Gynecology and Reproductive Medicine, University Women’s Hospital Jena, Jena University Hospital, Friedrich-Schiller-University, 07747 Jena, Germany

**Keywords:** Prognostic markers, Breast cancer

## Abstract

To investigate whether automated volumetric radiomic analysis of breast cancer vascularization (VAV) can improve survival prediction in primary breast cancer. 314 consecutive patients with primary invasive breast cancer received standard clinical MRI before the initiation of treatment according to international recommendations. Diagnostic work-up, treatment, and follow-up was done at one tertiary care, academic breast-center (outcome: disease specific survival/DSS vs. disease specific death/DSD). The Nottingham Prognostic Index (NPI) was used as the reference method with which to predict survival of breast cancer. Based on the MRI scans, VAV was accomplished by commercially available, FDA-cleared software. DSD served as endpoint. Integration of VAV into the NPI gave NPI_VAV_. Prediction of DSD by NPI_VAV_ compared to standard NPI alone was investigated (Cox regression, likelihood-test, predictive accuracy: Harrell’s C, Kaplan Meier statistics and corresponding hazard ratios/HR, confidence intervals/CI). DSD occurred in 35 and DSS in 279 patients. Prognostication of the survival outcome by NPI (Harrell’s C = 75.3%) was enhanced by VAV (NPI_VAV_: Harrell’s C = 81.0%). Most of all, the NPI_VAV_ identified patients with unfavourable outcome more reliably than NPI alone (hazard ratio/HR = 4.5; confidence interval/CI = 2.14-9.58; P = 0.0001). Automated volumetric radiomic analysis of breast cancer vascularization improved survival prediction in primary breast cancer. Most of all, it optimized the identification of patients at higher risk of an unfavorable outcome. Future studies should integrate MRI as a “gate keeper” in the management of breast cancer patients. Such a “gate keeper” could assist in selecting patients benefitting from more advanced diagnostic procedures (genetic profiling etc.) in order to decide whether are a more aggressive therapy (chemotherapy) is warranted.

## Introduction

Breast cancer is the most common malignant neoplasm of women in the western world. Despite substantial advances in diagnosis and treatment, morbidity and mortality of breast cancer remain high^1^. By adjusting the treatment to the individual cancer biology, *precision medicine* aims to both improve disease survival and to reduce the rate of unnecessary cytotoxic treatment^2,3^.

To achieve this goal, accurate *biomarkers* are essential tools for precision medicine. They help us to understand tumor biology and allow estimation of patient outcome^4^. The Nottingham Prognostic Index (NPI) is a well-established and widely used method for the prediction of survival of primary breast cancer. It is based on three classic *biomarkers* of breast cancer: tumor size, tumor grade, and the number of metastatic loco-regional lymph nodes^5–8^.

Breast MRI (MRI) is a functional imaging method that has been validated in clinical practice for over 30 years^9^. Objective semi-quantitative analysis of MRI by automated, FDA-cleared software has been intensely evaluated over the last decade and has been clinically established for several years^10–12^. As automated analysis of MRI provides objective measures of pathological tissue vascularization and quantifies tumor response to cytotoxic treatment, it can be considered a typical *imaging biomarker*^4^.

In the past several years, the feasibility of MRI as a prognostic biomarker has been demonstrated^11–19^. Hereby, breast MRI dynamically investigates the cancer in real-time within the *whole tumor* volume *in vivo*. This concept offers some advantages over traditional biomarkers which are typically evaluated on a *small tumor sample ex vivo*. To achieve a high prognostic accuracy, the complex imaging data of breast MRI has to be investigated by a dedicated method. This method of correlating the extensive MRI imaging data with outcome parameters is referred to as radiomic analysis^20^.

This study aimed to investigate whether automated volumetric radiomic analysis of breast cancer vascularization (VAV) can improve survival prediction in primary breast cancer.

## Methods

### Patients

This investigation was designed as a retrospective observational single-center study. Data were collected at an academic, tertiary care institution run by interdisciplinary breast cancer specialists from the departments of gynaecology, oncology, pathology, radiation oncology, and radiology. This interdisciplinary breast center is certified according to national quality management criteria. The local ethical review board approved this study and waived the necessity for informed consent.

The following inclusion criteria were applied

- Patients were recruited over a consecutive time period
- Indication for MRI: Pre-treatment staging of lesions rated as category IV and V according to the Breast Imaging Reporting and Data System (BI-RADS) upon mammography and/or ultrasound
- Histologically verified diagnosis of invasive breast cancer
- Treatment and follow-up at our center.
- No neoadjuvant chemotherapy performed.

In case of recurrent or *in situ* breast cancer and known malignant neoplasms other than breast cancer, patients were excluded due to potential bias on MRI enhancement results or on outcome data. In addition to image-based analyses, data on demographic characteristics (sex, age, date of diagnosis), clinicopathological features, and individual patient outcome were collected.

### Patient outcome

The end point was defined according to the “Standardized Definitions for Efficacy End Points in Adjuvant Breast Cancer Trials” document^21^. Disease specific death (DSD) was used as the primary end point. The latter is an established variant to “overall survival”^21,22^. For DSD only, “death from breast cancer” is considered as “event”. So different from OS, cases with either “death from non-breast cancer cause” or “death from unknown cause” were not considered, but were censored. The remaining patients were defined as showing “disease specific survival” (DSS).

The analysis of patient outcome was based on routine follow-up at our center after completion of treatment. Survival time was documented as the interval [months] between the initial diagnosis and the last follow-up. The minimum follow-up time after treatment was 27 months for inclusion into the DSS group. Classification of patient outcome was established by the medical record of the last follow-up. In order to achieve valid results upon survival analysis, a mean survival time of at least 80 months was specified^23^.

### Clinicopathological features

Board-certified pathologists investigated the clinicopathological features, including standard measures of tumor size and disease extension (T1-T3, T4a–T4d). The number of resected lymph nodes was documented and further classified as N0–N3^24^. Histological subtypes were specified as proposed by the World Health Organization Classification of Tumours^24^. Tumor aggressiveness was classified as *Grade 1 to 3* according to^25^. Further details on clinicopathological features, including those not relevant for the NPI, are provided within the supplementary material.

Percentage of tumor cells expressing steroid receptors (*estrogen/progesterone receptors*: ER/PR) was evaluated (cut-off 1% of positive cells)^24,26^. The expression of the HER2 (Human epidermal growth factor receptor) was analyzed as specified in^27^. Ki-67 was not generally recommended at date of study conception, and so it was not included in the present analysis. Further details on the clinicopathological features are provided within the supplementary material.

### Magnetic resonance imaging

For data acquisition, we used standard MRI protocols, with an overall examination time of 14 minutes. To achieve homogenous enhancement data, every patient was imaged with the same imaging protocol using two 1.5 Tesla whole-body magnetic resonance imaging scanners (Magnetom Symphony; Siemens Healthineers, Erlangen, Germany) and a dedicated, vendor-supplied, bilateral, four-channel breast coil.

This imaging protocol was in accordance with international recommendations^28^: For the present analysis, dynamic T1-weighted gradient-echo images only were investigated, with 60 seconds temporal resolution and an examination time of eight minutes (fast low angle shot/FLASH; flip angle 80°, repetition time 113 ms, echo time 5 ms, voxel size: 1.1 × 0.9, slice thickness 3 mm). This dynamic sequence was acquired before and after intravenous bolus injection (automatic injector: Spectris, Medrad, Pittsburgh, USA) of gadopentetate dimeglumine (Bayer HealthCare, Leverkusen, Germany) at a dosage of 0.1 mmol/kg body weight followed by 20 ml saline solution. A delay of 30 seconds after contrast medium application was set before post-contrast images were acquired under identical tuning conditions for a total of 7 measurements. Further technical details of the MRI protocol are provided within the supplementary material.

### Volumetric analysis of breast cancer vascularization (VAV)

Raw data of MRI were analyzed by automated, FDA-approved, commercially available software (BreVis, Siemens Healthineers, Erlangen, Germany). The software was operated by a reader blinded to clinicopathological features and patient outcome. The reader received special training in handling the software and was supervised by two radiologists with at least 7 years of experience in breast MRI. This kind of software has been scientifically evaluated since 2005 in multiple institutions, and has been in clinical use for several years. A detailed description can be found in the literature^10–12^.

**Figure 1:**
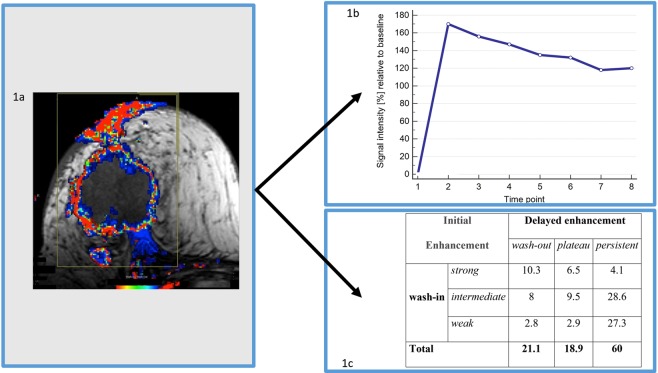
Clinical case illustrating the assessment of VAV. MRI of a 51 year old female patient (invasive ductal cancer, G2, diameter 5.8 cm, T4c, HER2 negative, hormonal receptor negative, 5 positive lymph nodes, NPI = 6.2). (**A**) *Heat map* of the color-coded CAD overlay of the T1 pre-contrast scan reflecting h*eterogeneity of vascularization*. The colors are coded from red to blue and reflect the wash-out ratio (see main text for definition). A large heterogeneously enhancing mass with infiltration of skin and thoracic wall and central necrosis is visualized, consistent with T4c. Note the discrepancy between the vital tumor assessed by MRI (color coded parts. TTV) and the larger morphologic extensions including the central necrosis. (**B**) Vascularization of the *most suspect tumor compartment. A* strong wash-in (170%) followed by a significant wash-out (50%) is demonstrated. The time to peak enhancement (TTP) was one minute. (**C**) Heterogeneity of vascularization summarized in a 3×3 matrix. There was a wide range of vascularisation patterns present within the same tumor. A relevant proportion of the TTV (10.3%) exhibited a pattern typical of hypervascularization and vascular shunting (category I: strong wash-in and wash-out). Nevertheless, the majority of the tumor demonstrated patterns indicative of a less pronounced neovascularization (weak or intermediate wash-in and persistent: 55.9% of the TTV). Both the NPI and the VAV were suggestive of a poor outcome. This patient died from breast cancer 18 months after initial diagnosis.

The only user-dependent step during analysis of MRI was defining the volume of interest. This was achieved by encircling a rectangular cuboid around the tumor (see Fig. 1)^29^. Based on our experience with the given protocol over many years, the minimum initial enhancement threshold was set to 30% (change of signal intensity first minute after contrast relative to baseline in [%]). As a consequence, perifocal tissue or necrotic tumor components were automatically excluded from further analysis. In case of multiple tumors per breast, the largest one was defined as index lesion.

Based on established dynamic enhancement patters^29^, the *vascularization pattern of every voxel* within the tumor was characterized. In total, the software automatically extracted 14 quantitative and semi-quantitative radiomic features for the VAV. These parameters assessed three aspects of tumor vascularization:

I. ***Total enhancing tumor volume (TTV)*** characterized the amount of vital cancer tissue and was measured as [cm^3^]. It was defined as the sum of tumor voxels surpassing the initial enhancement threshold (see above). Accordingly, the TTV addressed only the vital cancer tissue and did not consider areas of necrosis. Therefore, the TTV has also been called “functional tumor volume” previously (Table 1, parameter #1)^18^.

**Table 1:** Summary of parameters used for MRI analysis.

Parameter			Unit	Definition	
1	**Total tumor volume (TTV)**		cm^3^	Sum of tumor voxels with wash-in > 30%^§^	
	**Heterogeneity of vascularization**			**wash-in**	**delayed enhancement**
		**category:**			
2		**A**	% (of TTV)	weak (30% to 50%)	persistent
3		**B**			plateau
4		**C**			wash-out
5		**D**		intermediate (50% to 100%)	persistent
6		**E**			plateau
7		**F**			wash-out
8		**G**		strong (>100%)	persistent
9		**H**			plateau
10		**I**			wash-out
	**Most suspect tumor compartment**				
		**parameter:**			
11		**time to peak enhancement (TTP)**	minutes		
12		**peak enhancement**	%^§^	maximum contrast uptake by the tumor during the entire dynamic scan	
13		**wash-in**		contrast uptake by the tumor one minute after contrast application	
14		**wash-out ratio**	n.a.	ratio of wash-in and wash-out	

Note: The software investigates the signal intensity of every tumor voxel (size 1.1×0.9×3 mm^3^) during the dynamic MRI scan (one pre- and seven post-contrast scans at one-minute temporal resolution). Thus, the software calculated *11 quantitative* (parameters 1 to 11: [cm^3^] or [minutes]) and *three semi-quantitative* parameters (parameters 11 to 14). This enabled identification of the total tumor volume (TTV), volumetric analysis of tumor vascularization with a focus on tissue heterogeneity (VA: Parameters 2 to 10), and a detailed investigation of the most suspect tumor compartment (parameters 11 to 14)

**Notes: Wash-in**: Initial contrast uptake by the tumor during the first minute after contrast application. Values refer to the baseline signal before contrast administration. **Delayed enhancement**: Change of lesion signal behaviour between the seventh and first minute after contrast application. Three categories are defined: **persistent** (>10% further signal increase), **plateau** (stable signal ±10%) and **wash-out** (>10% signal decrease). ^**§**^Percentages normalized to baseline signal intensity before application of contrast media.
II. ***Heterogeneity of vascularization**** (within the TTV)*. Breast cancer is a heterogeneous disease and various vascularization patterns can be present in the same cancer in parallel. Accordingly, a wide range of enhancement patterns can be observed within the TTV^16^. This aspect was investigated by the *heterogeneity of vascularization*. Hereby, every voxel of the tumor was investigated as follows:
  Initially, the enhancement patterns within each voxel were identified following international breast MRI standards^29,30^. The initial enhancement phase investigates the first minute after contrast application. It was categorized as weak (30% to 50%), intermediate (50% to 100%), or strong “wash-in” (>100%), with percentages expressing the change of signal intensity during the first minute after contrast media application, relative to baseline^29,30^.
  The delayed enhancement phase investigates the change of contrast enhancement between the first and the last minute after contrast media application. It was categorized as persistent (>10%), plateau (+/−10%), or wash-out (−10%). Percentages express the change of signal intensity during the last minute after contrast media application, relative to the first postcontrast scan^29,30^.
  Combining the initial phase (n = 3) and delayed phase categories (n = 3) yielded nine enhancement patterns to classify the vascularization of each voxel within the TTV (for instance as “weak wash-in” and “persistent”, etc.).
  Finally, the tumor volume was subdivided according to these nine enhancement patterns. Hereby, the proportion relative to the TTV [%] was identified for every enhancement pattern (Table 1, parameters #2–10).
III. Vascularization of the ***most suspect tumor compartment***. In this analysis only one particular compartment of the entire tumor volume was investigated. For this reason, the TTV was divided into clusters (size: 3×3 voxels). Within every cluster, the ratio of the corresponding initial versus delayed enhancement was computed (wash-out ratio). According to the literature, the cluster achieving the highest wash-out ratio was defined as the “most suspicious tumor compartment”^11^.

The “most suspicious tumor compartment” was characterized by the following four radiomic features (Table 1, parameters #11–14): “wash-out ratio” (see above), “wash-in” (signal intensity first minute after contrast relative to baseline [%]), “peak enhancement” (maximum contrast uptake by the tumor during the whole dynamic scan), and time to “peak enhancement” (TTP [minutes]).

A clinical case illustrating the workflow of the volumetric analysis of breast cancer vascularization is given in Fig. 1. Table 1 summarizes all 14 parameters provided by the VAV.

### Statistical analysis

Disease specific death (DSD) was defined as the primary study endpoint. Association of clinicopathological features, patient age, NPI, and NPI_VAV_ with survival outcome was explored by cox regression and likelihood ratio tests.

All tests were two-sided. In this exploratory study, an alpha error below 10% was defined as appropriate to reject the null hypothesis. Considering the total number of variables (n = 14), we applied a conventional Bonferroni correction for alpha-error accumulation. Accordingly, the significance level was set to *P* = 0.007.

### NPI

According to^7^, the NPI was calculated as follows:

$${\rm{NPI}}=[0.2\times {\rm{S}}]+{\rm{N}}+{\rm{G}}$$

Hereby, S is the size of the index lesion in centimeters, N is the nodal status (0 nodes = 1; 1–4 nodes = 2;>4 nodes = 3), and G is the tumor grade (Grade I = 1; Grade II = 2; Grade III = 3).

The association of NPI with survival was investigated by cox regression, and predictive values were saved. The corresponding performance of NPI to predict DSD was validated by the Harrell’s C index. The latter is an extension of the area under the ROC curve for censored survival data^31^.

### VAV

Association of the 14 VAV parameters with the endpoint was analyzed by *univariate* cox regression.

In addition, *multivariate* analysis was performed. Only VAV revealing significant association with DSD upon univariate analysis were eligible for this step. Multivariate cox regression with backward feature selection was applied (*P* for enter/removal: 0.001/0.05). Feature selection removed redundant or irrelevant VAV. Furthermore, feature selection enabled simplification and – by reducing overfitting – generalization of the model. Corresponding predictive values of the multivariate model were saved and investigated by the Harrell’s C index as described for NPI.

### NPI_VAV_

NPI_VAV_ was designed as a compound measure of NPI and VAV. VAV revealing significant association with DSD upon univariate analysis and the NPI were eligible as covariates. In order to construct NPI_VAV_, multivariate cox regression with backward feature selection was applied as described above. Predictive values were saved. Performance of NPI_VAV_ to predict DSD was investigated as described for NPI (Harrell’s C index).

### Comparison of outcome prediction

Finally, predictions of DSD by NPI and VAV were compared. For this purpose, the corresponding predictive values by the cox regressions were compared^32^.

Kaplan Meier survival analysis was used to graphically illustrate differences between NPI_VAV_ and NPI. Hereby, the cut-off criterion for the prediction of DSD was optimized to the value that maximized Youden’s J statistic on the time-dependent data. For the latter, we used the predictive values of the corresponding cox regression analysis of NPI and NPI_VAV_^33,34^.

The differences between the corresponding Kaplan Meier survival curves were explored by hazard ratios (HR), corresponding confidence intervals (CI), and the logrank test^23^.

### Statement of human rights

All procedures performed in studies involving human participants were in accordance with the ethical standards of the institutional and/or national research committee and with the 1964 Helsinki declaration and its later amendments or comparable ethical standards. For this type of study formal consent is not required.

## Results

A detailed listing of background clinical data and clinicopathological factors is given in the supplementary material. This also contains a detailed uni- and multivariate analysis of study data including subgroup analysis.

### Characteristics of patients and subgroups

There were 314 patients with primary breast cancer who were included (DSS = 279; DSD = 35). Mean survival time was 84.6 months (CI: 81.9–87.2, standard error: 1.3). Range of follow-up interval was 27–93 months for DSS. On average, DSD occurred 27.9 months after initial diagnosis (range: 3–70 months).

*Mean patient age* was 57.6 years (SD: 11.7). Mean age within the DSS (mean: 57.4 years; SD: 11.7) and the DSD group was similar (mean: 59.6 years; SD: 11.5). Patient age could not be identified as a predictive parameter upon univariate analysis (*P* = 0.23). Inclusion of patient age into the NPI did not significantly increase prediction of DSD (*P* = 0.13). The same results were observed when including patient age into the NPI_VAV_ (*P* = 0.23).

### Clinicopathological features

Clinicopathological features showed expected distributions between the DSS and DSD group: There was a significant association with T- and N-Stage versus survival outcome (*P* < 0.001). Notably, the DSD exhibited larger tumors (e.g., T3: HR = 9.4; *P* < 0.0001) with a higher proportion of nodal-positive cases (e.g., N2: HR = 3.8; P = 0.0002). There was no significant association with Grading (*P* = 0.96) and histological subtypes (*P* > 0.77) on survival outcome. Key results of the clinicopathological features, stratified by the endpoints, are summarized in Table 2.

**Table 2:** Summary table of tumor characteristics.

Parameter		Outcome		Total
		DSS	DSD	
T-Stage	T1a	26	1	27
		9.30%	2.90%	8.60%
	T1b	43	3	46
		15.40%	8.60%	14.60%
	T1c	117	6	123
		41.90%	17.10%	39.20%
	T2	80	17	97
		28.70%	48.60%	30.90%
	T3	7	4	11
		2.50%	11.40%	3.50%
	T4	6	4	10
		2.20%	11.40%	3.20%
Histological subtype	Invasive ductal (not otherwise specified)	212	27	239
		76.00%	77.10%	76.10%
	Invasive lobular	23	4	27
		8.20%	11.40%	8.60%
	Mixed (Invasive lobular and ductal)	38	4	42
		13.60%	11.40%	13.40%
	Invasive medullary	3	0	3
		1.10%	0.00%	1.00%
	Invasive mucinous	3	0	3
		1.10%	0.00%	1.00%
Histological Grading	G1	12	0	12
		4.30%	0.00%	3.80%
	G2	108	13	121
		38.70%	37.10%	38.50%
	G3	159	22	181
		57.00%	62.90%	57.60%
Total		**279**	**35**	**314**
		**100.00%**	**100.00%**	**100.00%**

Note: tumor characteristics stratified by the clinical outcome given as DSD and DSS (disease specific survival and death).

^a^A more detailed overview of clinicopathological characteristics is given in the supplementary material.

### NPI

Mean NPI was 4.4 (95% CI = 4.3–4.5). In patients with DSD, the mean NPI (5.2; CI = 4.8–5.6) was significantly higher compared to the DSS group (4.3; CI = 4.1–4.4; P < 0.001). Harrell’s C was 75.3% for NPI. Descriptive statistics of the NPI are summarized in Table 3.

**Table 3:** Descriptive statistics of parameters retained in the NPI_VAV_ model and patient age.

Survival	Total tumor volume (TTV [cm^3^])		Heterogeneity of vascularization*		Time-to-peak enhancement (TTP [min])		NPI		Age [years]	
	DSS	DSD	DSS	DSD	DSS	DSD	DSS	DSD	DSS	DSD
**Minimum**	0.1	0.5	2.4	5.4	1	1	2.2	3.2	27	36
**Maximum**	313.1	249.7	80	45.3	4	6	7.2	7.5	87	82
**Mean**	7.1	29.9	29.3	23	1:18	1:42	4.3	5.2	57.4	59.6
**Median**	2.9	7.6	27	23.2	1	1	4.3	4.7	58	61
**SD**	21.1	60.4	14.1	8.6	0:42	1:12	1	1.2	11.7	11.5

Note: Given are all four parameters retained in the NPI_VAV_ model and patient age (see Table 4). Corresponding results of descriptive statistics are listed. Except patient age (*P* = 0.23), all parameters were predictive of the endpoint (for *P*-values, see Table 4). *Weak wash-in (initial phase) and persistent (delayed phase) [%] (details see Table 1 and main text).

### VAV

VAV revealed significant association with the endpoint: Time to peak enhancement (TTP: *P* = 0.003), total tumor volume [cm³] (*P* < 0.0001) and one parameter focusing on the heterogeneity of vascularization (category A: *P* = 0.006) were significant predictors of DSD. Predictive performance of VAV reached Harrell’s C = 74.0% and was equal to that of NPI (*P* = 0.92). Key VAV stratified by the endpoints are summarized in Table 3.

In addition, peak enhancement was slightly higher in the DSD group (DSD/DSS = 123.5/110%; *P* = 0.07), as was wash-in (DSD/DSS: 119.6/107.1%; *P* = 0.08). Both parameters, however, missed the significance level.

### NPI versus NPI_VAV_

Feature selection retained the NPI and all three parameters of VAV in the final NPI_VAV_ model (Table 4). Corresponding model fit was superior compared to NPI alone (Chi-squared: 51.5 vs. 27.9). Harrell’s C was 81.0% for NPI_VAV_ and surpassed the value for NPI by 5.6%.

**Table 4:** NPI_VAV_ model.

Covariate	HR	CI	*P*	Coefficient	SE
Total tumor volume (TTV)	1.01	1.00 to 1.01	0.04	0.01	0.00
Heterogeneity of vascularization*	0.95	0.92 to 0.98	0.002	−0.05	0.02
Time-to-peak enhancement (TTP)	1.84	1.40 to 2.42	<0.0001	0.61	0.14
Nottingham Prognostic Index (NPI)	2.01	1.48 to 2.73	<0.0001	0.70	0.16

Note: Given is the NPI_VAV_ model as provided by the Cox regression. After applying feature selection, four covariates were retained. Besides NPI this included three parameters for MRI analysis (for details, see Table 1).

**CI**: 95% confidence interval of the hazard ratio. **SE**: standard error of the coefficient. *Weak wash-in (initial phase) and persistent (delayed phase) [%] (details see Table 1 and main text).

**Figure 2:**
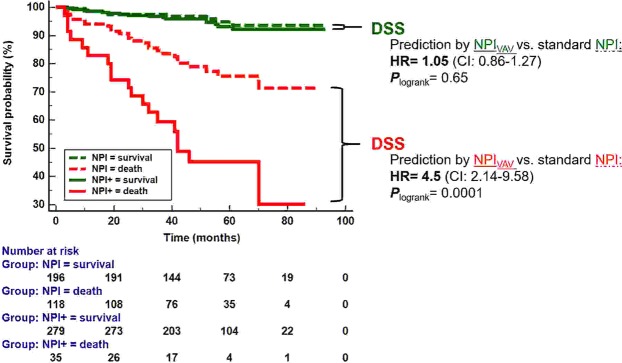
Prediction of good (DSS) and poor (DSD) patient outcome: Comparison of NPI_VAV_ vs. standard NPI. NPI_VAV_ enabled a better identification of patients at risk for DSD compared to standard NPI (**HR** = 4.5, CI: 2.14–9.58). This was also evident by a faster decline of the corresponding survival curve ***(P***_logrank_ = 0.0001). Optimized identification of high risk patients by the NPI_VAV_ did not come on the price of a worse identification of patients with a more favorable outcome. Indeed, the likelihood of **DSS** was alike if NPI_VAV_ was used to predict DSS, compared to standard NPI alone (HR = 1.05, CI: 0.86–1.27, ***P***_logrank_ = 0.65).

The improved survival prediction by VAV was also demonstrated by the Kaplan Meier analysis (Fig. 2):

#### Prediction of DSD

The survival curves of patients in whom NPI_VAV_ predicted DSD significantly differed from those in whom NPI alone predicted DSD (logrank test: *P* = 0.0001). DSD was more likely to occur, if this event was predicted by NPI_VAV_, compared to NPI alone (HR = 4.5; CI = 2.14–9.58).

#### Probability of DSS

The survival curves of patients classified as DSS either by NPI_VAV_ or by NPI were not significantly different (logrank test: *P* = 0.65). The risk of DSS was similar, if DSS was predicted by NPI_VAV_, compared to the patients in whom NPI alone predicted DSD (HR = 1.05; CI: 0.86–1.27).

## Discussion

Breast MRI contributed to the prediction of survival in patients with primary invasive breast cancer. Automated volumetric radiomic analysis of breast cancer vascularization improved survival prediction compared to the Nottingham Prognostic Index alone (NPI). The NPI is a well-established and widely used method of predicting survival of primary breast cancer. Most of all, the use of MRI optimized the stratification of patients at risk of an unfavourable survival outcome

### MRI showed a prognostic accuracy equal to that of the NPI

To our knowledge, we are the first group to report a head-to-head comparison of MRI and NPI in the risk stratification of patients. One advantage of prognostic MRI is that it is performed as a *one-stop-shop procedure* within one session. Our approach was based on a standard MRI protocol, as recommended by international guidelines, and required only 14 minutes of magnet time^28^. Based on this fast examination, both the staging — including the assessment of tumor extension plus multifocal and bilateral disease — and the risk estimation can be accomplished. Whereas classical risk factors such as nodal metastasis and histological grading are nominal or ordinal by nature, MRI provides *quantitative and semi-quantitative* parameters. This information is semi-*automatically assessed by the software*, which is an advantage over standard pathological assessment. For instance, the evaluation of grading is known to be limited by significant inter-pathologist variability^35^. *Prognostic MRI information moreover is available in real-time*. This is a potential advantage for clinical workflow and patient management. Of note, however, some NPI cannot be assessed on core biopsy samples, but require surgical resection for definitive classification. This is frequently the case in nodal staging, resulting in the delayed availability of these biomarkers for prognostic purposes. MRI both *volumetrically and dynamically investigates the whole tumor volume in vivo*. This is an advantage over most prognostic biomarkers that evaluate small tumor samples *ex vivo*. As MRI is *not a snapshot diagnosis*, it is well suited to investigate tumor heterogeneity. In fact, volumetric MRI features were among the best prognostic parameters in our analysis.

### Our findings support the evaluation of MRI as a prognostic biomarker in precision medicine

The concept of precision medicine aims to adapt cancer treatments to an individual’s tumor characteristics. Thus, the goal of precision medicine is to both maximize the effectiveness of treatment and to minimize the side effects of cytotoxic drugs^2^. As every known biomarker thus far has limitations, there is intensive ongoing research into the development of new tissue biomarkers. One of the most promising fields is gene expression profiling of breast cancer^2,3,36,37^. Gene expression profiling is already used in systemic breast cancer treatment planning^3^, and empiric evidence shows that the decision towards more aggressive treatments such as cytotoxic drugs can be based on distinct gene profiles^37^. MRI could actually tread the same path: In our analysis, the strength of MRI was a more accurate identification of patients with a less favorable outcome. Thus, our data could be used to stratify patient risk. Certainly, it would be unwise to apply a more aggressive treatment based only on the MRI information at the time being. Nevertheless, MRI might be used as a “gate keeper”. If MRI indicates an unfavorable outcome, further biomarkers such as genetic profiling might be warranted, in order to decide whether a more aggressive therapy is indicated.

### Possible biological correlates for our MRI findings will be discussed

Tumor size is one of the best-established prognostic parameters of breast cancer^38^. Typically, the measurement of the breast cancer size is performed as linear measurements in two spatial dimensions. Both the NPI and the T-staging are based on this method^5,8,25^. The breast carcinoma may grow in a diffuse pattern, as is often observed in invasive lobular subtypes^39^. Consequently, the tumor size of breast cancer cannot always be accurately measured by two-dimensional linear measurement. However, TTV is a volumetric method and thus can evaluate the tumor size in any spatial extent.

However, TTV is not only a metric parameter of tumor size. According to the research by Hylton *et al*. it can also be interpreted as a functional biomarker of vascularization^18^. Neoangiogenesis is regarded as a pivotal step in the development of cancer^40^. At the same time, neoangiogenesis significantly determines the potential of a breast cancer to metastasize and hereby limits patient outcome^41,42^. One method to quantify tumor vascularization is the determination of its microvascular density (MVD). It is therefore conclusive that microvascular density can also be considered as a prognostic biomarker of breast cancer^42^. Interestingly, contrast enhancement assessed by MRI was closely linked to MVD in previous radiopathological correlations studies^43^. Thus, the prognostic significance of TTV may also be explained in this functional context. However, tumor size alone is not a sufficient parameter to optimally predict the prognosis of a patient. This explains why additional parameters were identified as independent predictors of survival status in the NPI_VAV_ model^44^.

Different from the classical pathological analysis as well as from modern methods (such as genetic profiling), MRI can examine the entire tumor volume^16,19,25,36,45^. For this reason, the method can also be used to assess the intratumoral heterogeneity. The latter is a major aspect in breast cancer biology^46^. We identified “heterogeneity of vascularization” within the tumor as a significant parameter of patient outcome. Hereby, a higher volume of compartments exhibiting slow wash-in and persistent enhancement corresponded to a more favorable prognosis. Of note, this MRI pattern has been described by Leong *et al*. as typical for breast cancers^45^. Considering the relationship of MRI enhancement and the MVD (see above), this finding seems conclusive: Buadu reported that breast cancer with slower initial and delayed contrast enhancement in MRI exhibited lower MVD in radiopathological correlation^43^. Since a lower MVD is regarded as a predictor of a relatively better outcome, this finding seems conclusive^42^.

The analysis of tumor heterogeneity also enabled the identification of the most suspicious compartment. It was identified based on the parameters wash-in and wash-out. In the context of the aforementioned MVD, both parameters could be regarded as parameters of tissue vascularisation^42,43^. Within this compartment, a slightly faster wash-in, a slightly higher maximum enhancement, and, above all, a significantly longer TTP were observed in the DSD group. Wash-in can be interpreted as a parameter of tissue perfusion^47^. It reflects both the blood flow and the intravascular blood volume (for instance figure 5 in^47^), and correlates with the MVD^42,43,47^. After the perfusion phase, the contrast medium is transmitted through the blood vessels into the interstitium. This event is referred to as leakage and here the maximum enhancement is typically observed^47^. However, it needs to be emphasized that an exact separation of the different pharmacokinetic compartments is not feasible with standard breast MRI protocols^47^. Leakage is not only determined by the quantity (vessel surface), but also by the quality (permeability) of the vessels^47^. The quantity (vessel surface) correlates with the MVD^41,47^. The permeability is triggered by numerous factors, among others by VEGF^48^. VEGF is a cytokine that plays a central role in the process of angiogenesis^48^. These considerations might explain why the maximum enhancement in the DSD group was slightly higher and slightly delayed compared to the DSS cases. Interestingly, the parameter TTP is rarely used in breast MRI diagnostics. In different fields of radiology, such as in neuro imaging, the TTP is a standard parameter^47^. This is particularly relevant since early breast MRI research has already shown that TTV is superior to more commonly used qualitative kinetic criteria^49^. In this context, an in-depth re-evaluation of this parameter may be of promise.

### Some limitations of our work will be discussed

The literature reports a large number of breast cancer biomarkers. Hereby, the NPI was introduced in 1982 and remains a widely used method to predict survival in primary breast cancer^5,6,8^. A completely different approach is taken by multigene assays. These rather new biomarkers focus on genetic analysis and are increasingly used in clinical routine^50^. Interestingly, it has already been shown in the literature that there is a close correlation between MRI parameters with multigene assays such as MammaPrint, Oncotype DX, and PAM50^51^. So future studies might investigate to what extent MRI analysis could substitute genetic tests, or whether MRI might actually improve the prognostic information provided by multigene assays.

We investigated radiomic parameters of tumor vascularization. We excluded morphologic features from our analysis^20^. Previous studies have shown that also morphologic MRI data provide prognostic information^17,52^. The presence of avascular, seemingly necrotic compartments have for instance been associated with surrogates of poor outcome^52^. So future studies should investigate textural radiomic features as well in order to determine whether predictive accuracy of VAV can be further increased.

Breast cancer can metastasize after many years and lead to DSD^53^. It cannot be excluded that some of our patients died after the end of data collection. This is a potential bias of the study.

We conceptualized this work as a cross-sectional study. In this respect, our data represent a typical patient collective at our practice. Therefore - as well as in the context of the patient number - no formal subgroup analysis according to histopathological parameters (clinicopathological factors, etc.) as well as the treatment regime was performed. Therefore, we cannot exclude that these two parameters had an impact on our results. In the same way, we decided to exclude patients with NAC from the analysis. These patients were predominantly included in clinical trials and were therefore treated with a therapy not yet established. Nevertheless, this implies a potential limitation of our study: Results do not apply on NAC patients.

In distinction to the classical visual MRI analysis, the influence of the reader on the VAV is significantly lower^54^. The software works largely automated and the only investigator-dependent step is the definition of the volume of interest. Nevertheless, some observer-dependent bias within our data cannot be excluded. Furthermore, the reproducibility of our results is also influenced by the software itself. Although the software is established on the market, our results might not be completely reproducible on other systems. Finally, the MRI protocol itself can also have influence on our results^55^. We aimed to control for this bias and adopted our imaging protocol to international standards and used well established criteria to assess the tumor vascularization^16,18,28,29,45,56^. Future studies should investigate the repeatability of our results with different software, MRI protocols and identify the potential impact of the reader on our results.

## Conclusion

In conclusion, automated volumetric radiomic analysis of breast cancer vascularization improved the prediction of survival of patients with primary invasive breast cancer. Most of all it improved the identification of patients at higher risk of an unfavorable outcome.

These results are based on a standard, clinical MRI followed by real-time analysis by a commercially available, FDA-cleared computer algorithm. Accordingly, the method can be incorporated into the clinical routine.

One potential role of MRI would be a “gate keeper”, in order to identify patients requiring a more advanced diagnosis (genetic profiling etc.) and/or more aggressive therapies (chemotherapy). This approach should be evaluated in future trials.

## Supplementary information

supplementary material.

## Data Availability

All relevant data are within the paper and its supplementary files.
