# Supplementary material for: Automated volumetric radiomic analysis of breast cancer vascularization improves survival prediction in primary breast cancer
Source: Sci Rep. 2020 Feb 28;10:3664. doi: 10.1038/s41598-020-60393-9 (PMC7048934; doi:10.1038/s41598-020-60393-9)
Supplement: Supplementary file 1 — supplementary material. [file 41598_2020_60393_MOESM1_ESM.pdf]

Title Page

Automated volumetric radiomic analysis of breast cancer vascularization improves survival prediction in primary breast cancer

Shortened title: Automated radiomic analysis of breast cancer

Authors (first and last)

Matthias Dietzel and Pascal A.T. Baltzer*

Co authors

Rüdiger Schulz-Wendtland

Stephan Ellmann

Ramy Zoubi,

Evelyn Wenkel,

Matthias Hammon

Paola Clauser,

Michael Uder,

Ingo B. Runnebaum

Content

Content 1

Details on Patients and Methods 2

Assessment of clinicopathological factors (CPF) 2

Treatment of patients 4

Magnetic resonance imaging protocol 6

Univariate analysis: Detailed results 7

Clinicopathological factors 7

T-Stage 7

N-Stage 9

Typing 10

Additional DCIS 11

Grading 12

Estrogen receptors 13

Progesteron receptors 14

HER2 15

Molecular subtypes 16

Luminal A and B (without her2 enriched subtype) 16

Luminal B: her2 enriched subtype 17

Her2 enriched subtype 18

Basal like 19

Breast MRI parameters 20

VAV: vascularization of the most suspect tumor compartment 20

Volumetric analysis: Heterogeneity of vascularization 21

Multivariate analysis: VAV 24

References 25

Details on Patients and Methods

Assessment of clinicopathological factors (CPF)

T-Stage: Tumor resection was performed by a surgical gynaecologist. After formalin *fixation* the surgical specimen was examined by a breast pathologist: T-Stage (1 to 3) was assigned based on the largest diameter of the tumor as measured on representative slices. In case of chest wall/skin invasion or inflammatory cancer T-4 Stage was diagnosed [1,2].

N-Stage: If lymph nodes were clinically or pathologically positive, axillary node dissection was carried out by a surgical gynaecologist. In all other cases, the sentinel method was standard of care. It was performed subsequent to subareolar injection of technetium 99m Sulfur Colloid and blue dye. The resulting surgical specimen were formalin *fixed and* evaluated by the pathologist: Hereby eight representative sections were chosen, paraffin-embedded and stained with haematoxylin and eosin (HE). Then, all sections were assessed with conventional microscopic examination. In case of unclear results, immunohistochemistry was performed using dedicated Pan-Cytokeratin antibodies (AE1/AE3, MNF-116). Finally, presence of metastatic deposits was staged according to the TNM system. Additionally, the numbers of resected and affected nodes were documented in the database [1,2].

Typing: Following paraffin-embedding and *haematoxylin* staining of the surgical specimen, defined field areas of representative slices where examined. Based on standard histo-morphological criteria assessed with conventional light microscopy, tumors were dichotomized as “in situ disease” (basal membrane intact; exclusion criteria - see material and methods) or “invasive cancer” [1,2]. According to the WHO criteria invasive carcinomas where further classified as “ductal”, “lobular”, “mixed type” to name only the most common diagnoses. In case of doubt appropriate additional stainings where performed [1].

Grading was done on defined field areas of representative HE stained slices. For grading, the percentage of tubule formation, the degree of nuclear pleomorphism and mitotic count within a given field of view are assessed. Based on the Elston-Ellis method it allows to morphologically assess the degree of tumor differentiation based on a three-stage qualitative score as Grade 1 to 3 (G1 to 3) [3].

Steroid receptors: Immunohistochemical staining of estrogen (ER: Dako Monoclonal Mouse Anti Human Estrogen Receptor α Clone 1D5) and progesterone receptors (PR: Dako Monoclonal Mouse Anti Human Progesterone Receptor Clone PgR 636) was performed adhering to a standardized protocol using formaldehyde-fixed, deparaffinised tissue sections. The percentage of positive cells was determined, resulting in a semi-quantitative measure for the ER and PR status [2,4].

Human epidermal growth factor receptor 2 (HER2): Based on deparaffinised tissue sections, immunohistochemical staining (Hercept TestTM for the Dako Autostainer Code K 5207) were conducted. Results were qualitatively rated on an ordinal score (0, 1+: positive, 2+, 3+: positive). Fluorescence in situ hybridization (FISH) tests were used to verify positive results of qualitative scores [5].

Treatment of patients

Patients were managed according to applicable national guidelines and current evidence from clinical trials [6,7]. In the following, criteria of the guidelines are summarized and complemented by the number of patients fulfilling the given criteria:

Every patient received *surgical resection* of the primary tumor. Hereby local control of disease was primarily gained by breast conserving therapy. Modified radical mastectomy was the treatment of choice in patients with locally advanced breast cancer at time of initial diagnosis (n=22).

Primary method for *surgical axillary staging* was sentinel node biopsy. It was performed after preoperative sub-areolar injection of technetium 99m sulfur colloid and blue dye. If either the sentinel lymph node biopsy was positive or in case of already clinically positive nodes, axillary lymph node resection was performed (n= 100). Hereby at least 10 nodes in Level I or II were resected (median 22, range 10–44).

Following surgical treatment, patients were routinely scheduled for breast irradiation. The typical procedure after breast conserving therapy covered the whole ipsilateral breast and the chest wall. According to the guidelines, a total dose of 50 Gy was applied in a regimen of 5 × 2.0 Gy cycles per week. Similarly, in patients eligible for radical mastectomy (n=22), irradiation therapy was indicated; hereby the irradiation field covered the ipsilateral chest wall. Note that all of these patients also had a significant axillary tumor load with a median of 15 metastatic nodes (range 5–42).

As recommended by national guidelines, cytotoxic *chemotherapy* was considered according to the individual patient risk factors. According to the study design, no patient received *neoadjuvant systemic chemotherapy*. Applicable criteria were HER2-positive cancers (n=89), estrogen and/or progesterone negative cancers (n=43), nodal-positive disease (n= 100), high tumor grade (G3: n=189), or young age <35 years (n=3).

The 89 patients with HER2-positive cancers were treated with Trastuzumab according to the current evidence from clinical trials. Patients positive for estrogen and/or progesterone receptors received endocrine treatment after the completion of the chemotherapy normally over 5 years.

Note: As the aim of this study was an interindividual comparison, the treatment regimen was not considered as a possible confounder. Therefore no more details on treatment were documented in the database.

Magnetic resonance imaging protocol

Patients were placed in the prone position and a venous access was placed in a cubital vein. The DCE-MRI protocol consisted of repetitive radiofrequency spoiled dynamic T1-weighted gradient echo sequences (Fast Low Angle SHot, FLASH 2D), acquired at 1-minute intervals over eight minutes. After one baseline scan, Gadolinium pentetate (Magnevist, Bayer/Schering HealthCare, Leverkusen, Germany) was administered as a venous injection at a dosage of 0.2 ml/kg body weight, followed by a 20 ml saline flush. A constant flow rate of 3 ml/sec was ensured by using an automated injector (Spectris, Medrad, Pittsburgh, USA). Postcontrast scanning started 30 seconds after contrast material injection using the same adjustment settings. Acquisition parameters for DCE-MRI were 110ms (repetition time), 5ms (echo time), 80° (flip angle), $1.1*0.9*3mm^3$ (spatial resolution) and 350 mm (field of view).

Univariate analysis: Detailed results

Clinicopathological factors

T-Stage

Cox regression

Covariate	b	SE	Wald	P	Exp(b)	95% CI of Exp(b)
T2	1,3517	0,3997	11,4330	0,0007	3,8639	1,7721 to 8,4248
T3	2,2478	0,5933	14,3526	0,0002	9,4672	2,9768 to 30,1093
T4	2,5216	0,5956	17,9255	<0,0001	12,4491	3,8971 to 39,7675

Note: Reference category = T1

Cross tabs

—			Survival		Total
			DSS	DSD	
T-Stage		Count	26	1	27
	T1a	% within T-Stage	96,3%	3,7%	100,0%
		**% within Survival**	9,3%	2,9%	8,6%
		% of Total	8,3%	,3%	8,6%
	T1b	Count	43	3	46
		% within T-Stage	93,5%	6,5%	100,0%
		**% within Survival**	15,4%	8,6%	14,6%
		% of Total	13,7%	1,0%	14,6%
	T1c	Count	117	6	123
		% within T-Stage	95,1%	4,9%	100,0%
		**% within Survival**	41,9%	17,1%	39,2%
		% of Total	37,3%	1,9%	39,2%
	T2	Count	80	17	97
		% within T-Stage	82,5%	17,5%	100,0%
		**% within Survival**	28,7%	48,6%	30,9%
		% of Total	25,5%	5,4%	30,9%
	T3	Count	7	4	11
		% within T-Stage	63,6%	36,4%	100,0%
		**% within Survival**	2,5%	11,4%	3,5%
		% of Total	2,2%	1,3%	3,5%
	T4	Count	6	4	10
		% within T-Stage	60,0%	40,0%	100,0%
		**% within Survival**	2,2%	11,4%	3,2%
		% of Total	1,9%	1,3%	3,2%
Total		Count	279	35	314
		% within T-Stage	88,9%	11,1%	100,0%
		% within Survival	100,0%	100,0%	100,0%
		% of Total	88,9%	11,1%	100,0%

N-Stage

Cross tabs

			Survival		Total
			DSS	DSD	
N-Stage	N0	Count	206	14	220
		% within N-Stage	93,6%	6,4%	100,0%
		**% within Survival**	73,8%	40,0%	70,1%
		% of Total	65,6%	4,5%	70,1%
	N1	Count	69	19	88
		% within N-Stage	78,4%	21,6%	100,0%
		**% within Survival**	24,7%	54,3%	28,0%
		% of Total	22,0%	6,1%	28,0%
	N2	Count	4	2	6
		% within N-Stage	66,7%	33,3%	100,0%
		**% within Survival**	1,4%	5,7%	1,9%
		% of Total	1,3%	,6%	1,9%
Total		Count	279	35	314
		% within N-Stage	88,9%	11,1%	100,0%
		% within Survival	100,0%	100,0%	100,0%
		% of Total	88,9%	11,1%	100,0%

Cox regression

Covariate	b	SE	Wald	P	Exp(b)	95% CI of Exp(b)
Nodal_Stage=1	1,3237	0,3526	14,0925	0,0002	3,7575	1,8892 to 7,4735
Nodal_Stage=2	1,7442	0,7571	5,3080	0,0212	5,7215	1,3073 to 25,0412

Note: Reference category = N0

Typing

Cross tabs

			Survival		Total
			DSS	DSD	
Typing	Invasive ductal	Count	212	27	239
		% within Typing	88,7%	11,3%	100,0%
		**% within Survival**	76,0%	77,1%	76,1%
		% of Total	67,5%	8,6%	76,1%
	Invasive lobular	Count	23	4	27
		% within Typing	85,2%	14,8%	100,0%
		**% within Survival**	8,2%	11,4%	8,6%
		% of Total	7,3%	1,3%	8,6%
	Mixed (Invasive lobular and ductal)	Count	38	4	42
		% within Typing	90,5%	9,5%	100,0%
		**% within Survival**	13,6%	11,4%	13,4%
		% of Total	12,1%	1,3%	13,4%
	Invasive medullary	Count	3	0	3
		% within Typing	100,0%	,0%	100,0%
		**% within Survival**	1,1%	,0%	1,0%
		% of Total	1,0%	,0%	1,0%
	Invasive mucinous	Count	3	0	3
		% within Typing	100,0%	,0%	100,0%
		**% within Survival**	1,1%	,0%	1,0%
		% of Total	1,0%	,0%	1,0%
Total		Count	279	35	314
		% within Typing	88,9%	11,1%	100,0%
		**% within Survival**	100,0%	100,0%	100,0%
		% of Total	88,9%	11,1%	100,0%

Cox regression

Covariate	b	SE	Wald	P	Exp(b)	95% CI of Exp(b)
Invasive lobular	0,1545	0,5375	0,08267	0,7737	1,1671	0,4092 to 3,3287
Mixed (Invasive lobular and ductal)	-0,03527	0,5370	0,004313	0,9476	0,9653	0,3388 to 2,7507
Invasive medul-lary	-11,5137	337,8354	0,001162	0,9728	0,0000	0,0000 to 127E+279
Invasive mucin-ous	-11,5125	305,6286	0,001419	0,9700	0,0000	0,0000 to 67,5E+252

Note: Reference category = Invasive ductal

Additional DCIS

Cross tabs

			Survival		Total
			DSS	DSD	
Additional DCIS	absent	Count	171	22	193
		% within Additional DCIS	88,6%	11,4%	100,0%
		**% within Survival**	61,3%	62,9%	61,5%
		% of Total	54,5%	7,0%	61,5%
	present	Count	108	13	121
		% within Additional DCIS	89,3%	10,7%	100,0%
		**% within Survival**	38,7%	37,1%	38,5%
		% of Total	34,4%	4,1%	38,5%
Total		Count	279	35	314
		% within Additional DCIS	88,9%	11,1%	100,0%
		% within Survival	100,0%	100,0%	100,0%
		% of Total	88,9%	11,1%	100,0%

Cox regression

Covariate	b	SE	Wald	P	Exp(b)	95% CI of Exp(b)
DCIS= present	-0,1199	0,3503	0,1172	0,7321	0,8870	0,4480 to 1,7563

Grading

Cross tabs

			Survival		Total
			DSS	DSD	
Grading	G1	Count	12	0	12
		% within Grading	100,0%	,0%	100,0%
		**% within Survival**	4,3%	,0%	3,8%
		% of Total	3,8%	,0%	3,8%
	G2	Count	108	13	121
		% within Grading	89,3%	10,7%	100,0%
		**% within Survival**	38,7%	37,1%	38,5%
		% of Total	34,4%	4,1%	38,5%
	G3	Count	159	22	181
		% within Grading	87,8%	12,2%	100,0%
		**% within Survival**	57,0%	62,9%	57,6%
		% of Total	50,6%	7,0%	57,6%
Total		Count	279	35	314
		% within Grading	88,9%	11,1%	100,0%
		% within Survival	100,0%	100,0%	100,0%
		% of Total	88,9%	11,1%	100,0%

Cox regression

Co-variate	b	SE	Wald	P	Exp(b)	95% CI of Exp(b)
G2	12,4636	240,9781	0,002675	0,9588	258739,1467	0,0000 to 310E+207
G3	12,6414	240,9780	0,002752	0,9582	309100,3403	0,0000 to 370E+207

Note: Reference category =G1

Estrogen receptors

Cross tabs

			Survival		Total
			DSS	DSD	DSS
estrogen receptors	negative	Count	40	12	52
		% within estrogen receptors	76,9%	23,1%	100,0%
		**% within survival**	14,3%	34,3%	16,6%
		% of Total	12,7%	3,8%	16,6%
	positive	Count	239	23	262
		% within estrogen receptors	91,2%	8,8%	100,0%
		**% within survival**	85,7%	65,7%	83,4%
		% of Total	76,1%	7,3%	83,4%
Total		Count	279	35	314
		% within estrogen receptors	88,9%	11,1%	100,0%
		% within survival	100,0%	100,0%	100,0%
		% of Total	88,9%	11,1%	100,0%

Cox regression

Covariate	b	SE	Wald	P	Exp(b)	95% CI of Exp(b)
estrogen receptors: positive	-1,0622	0,3565	8,8787	0,0029	0,3457	0,1725 to 0,6928

Progesteron receptors

Cross tabs

			Survival		Total
			DSS	DSD	DSS
progesterone receptors	negative	Count	45	12	57
		% within progesterone receptors	78,9%	21,1%	100,0%
		**% within survival**	16,1%	34,3%	18,2%
		% of Total	14,3%	3,8%	18,2%
	positive	Count	234	23	257
		% within progesterone receptors	91,1%	8,9%	100,0%
		**% within survival**	83,9%	65,7%	81,8%
		% of Total	74,5%	7,3%	81,8%
Total		Count	279	35	314
		% within progesterone receptors	88,9%	11,1%	100,0%
		**% within survival**	100,0%	100,0%	100,0%
		% of Total	88,9%	11,1%	100,0%

Cox regression

Covariate	b	SE	Wald	P	Exp(b)	95% CI of Exp(b)
Progesterone receptors: positive	-0,8314	0,3565	5,4404	0,0197	0,4354	0,2165 to 0,8757

HER2

Cross tabs

			Survival		Total
			DSS	DSD	DSS
HER2	negative	Count	140	13	153
		% within HER2	91,5%	8,5%	100,0%
		**% within survival**	50,2%	37,1%	48,7%
		% of Total	44,6%	4,1%	48,7%
	positive	Count	139	22	161
		% within HER2	86,3%	13,7%	100,0%
		**% within survival**	49,8%	62,9%	51,3%
		% of Total	44,3%	7,0%	51,3%
Total		Count	279	35	314
		% within HER2	88,9%	11,1%	100,0%
		% within survival	100,0%	100,0%	100,0%
		% of Total	88,9%	11,1%	100,0%

Cox regression

Covariate	b	SE	Wald	P	Exp(b)	95% CI of Exp(b)
HER2: positive	0,4622	0,3499	1,7451	0,1865	1,5875	0,7997 to 3,1517

Molecular subtypes

Luminal A and B (without her2 enriched subtype)

Cross tabs

			Survival		Total
			DSS	DSD	DSS
Luminal A and B	no	Count	152	26	178
		% within Luminal A and B	85,4%	14,6%	100,0%
		**% within survival**	54,5%	74,3%	56,7%
		% of Total	48,4%	8,3%	56,7%
	yes	Count	127	9	136
		% within Luminal A and B	93,4%	6,6%	100,0%
		**% within survival**	45,5%	25,7%	43,3%
		% of Total	40,4%	2,9%	43,3%
Total		Count	279	35	314
		% within Luminal A and B	88,9%	11,1%	100,0%
		% within survival	100,0%	100,0%	100,0%
		% of Total	88,9%	11,1%	100,0%

Cox regression

Covariate	b	SE	Wald	P	Exp(b)	95% CI of Exp(b)
Luminal A and B	-0,7825	0,3867	4,0933	0,0431	0,4573	0,2143 to 0,9759

Luminal B: her2 enriched subtype

Cross tabs

			Survival		Total
			DSS	DSD	DSS
Luminal B: her2 enriched subtype	no	Count	153	17	170
		% within Luminal B: her2 enriched subtype	90,0%	10,0%	100,0%
		**% within survival**	54,8%	48,6%	54,1%
		% of Total	48,7%	5,4%	54,1%
	yes	Count	126	18	144
		% within Luminal B: her2 enriched subtype	87,5%	12,5%	100,0%
		**% within survival**	45,2%	51,4%	45,9%
		% of Total	40,1%	5,7%	45,9%
Total		Count	279	35	314
		% within Luminal B: her2 enriched subtype	88,9%	11,1%	100,0%
		% within survival	100,0%	100,0%	100,0%
		% of Total	88,9%	11,1%	100,0%

Cox regression

Covariate	b	SE	Wald	P	Exp(b)	95% CI of Exp(b)
Luminal B: her2 enriched subtype	0,1852	0,3382	0,2997	0,5841	1,2034	0,6223 to 2,3273

Her2 enriched subtype

Cross tabs

			Survival		Total
			DSS	DSD	DSS
HER2_enriched	no	Count	266	31	297
		% within HER2_enriched	89,6%	10,4%	100,0%
		**% within survival**	95,3%	88,6%	94,6%
		% of Total	84,7%	9,9%	94,6%
	yes	Count	13	4	17
		% within HER2_enriched	76,5%	23,5%	100,0%
		**% within survival**	4,7%	11,4%	5,4%
		% of Total	4,1%	1,3%	5,4%
Total		Count	279	35	314
		% within HER2_enriched	88,9%	11,1%	100,0%
		% within survival	100,0%	100,0%	100,0%
		% of Total	88,9%	11,1%	100,0%

Cox regression

Covariate	b	SE	Wald	P	Exp(b)	95% CI of Exp(b)
HER2 enriched	0,9436	0,5327	3,1379	0,0765	2,5691	0,9093 to 7,2591

Basal like

Cross tabs

			Survival		Total
			DSS	DSD	DSS
basal like	no	Count	266	31	297
		% within basal like	89,6%	10,4%	100,0%
		**% within survival**	95,3%	88,6%	94,6%
		% of Total	84,7%	9,9%	94,6%
	yes	Count	13	4	17
		% within basal like	76,5%	23,5%	100,0%
		**% within survival**	4,7%	11,4%	5,4%
		% of Total	4,1%	1,3%	5,4%
Total		Count	279	35	314
		% within basal like	88,9%	11,1%	100,0%
		% within survival	100,0%	100,0%	100,0%
		% of Total	88,9%	11,1%	100,0%

Cox regression

Covariate	b	SE	Wald	P	Exp(b)	95% CI of Exp(b)
basal like	0,8319	0,5315	2,4502	0,1175	2,2977	0,8151 to 6,4770

Breast MRI parameters

VAV: vascularization of the most suspect tumor compartment

Cross tabs

Parameter	Subgroup	Mean	SD	Range	
**Peak enhancement**	all	111.5	44.7	31	354
	DSS	110.0	44.5	31	354
	DSD	123.5	44.7	43	228
**TTP**	all	1.4	0.8	1	6
	DSS	1.3	0.7	1	4
	DSD	1.7	1.2	1	6
**Wash-in**	all	108.5	43.2	19	291
	DSS	107.1	43.1	19	291
	DSD	119.6	43.1	36	224
**Wash-out**	all	39.4	23.3	-22	177
	DSS	39.0	22.5	-11	177
	DSD	42.5	28.9	-22	121
**Washout ratio**	all	2.5	4.9	0.9	70
	DSS	2.6	5.2	0.9	70
	DSD	2.1	1.7	0.9	9

Cox regression

Covariate	b	SE	Wald	P	Exp(b)	95% CI of Exp(b)
peak enhancement	0.01	0.00	3.27	0.07	1.01	0.9995 to 1.0129
TTP	0.48	0.16	8.91	0.003	1.61	1.1783 to 2.2076
Wash-in	0.01	0.00	3.03	0.08	1.01	0.9992 to 1.0138
Wash-out	0.00	0.01	0.57	0.45	1.01	0.9920 to 1.0181
Washout ratio	-0.04	0.07	0.36	0.55	0.96	0.8268 to 1.1066

Volumetric analysis: Heterogeneity of vascularization

Cross tabs

Parameter	Subgroup	Mean	SD	Range	
Fast wash-in and persistent	all	2.8	5.0	0	68
	DSS	2.7	5.2	0	68
	DSD	3.8	3.8	0	16
Intermediate wash-in and persistent	all	23.3	10.3	0	57.1
	DSS	23.4	10.4	0	57.1
	DSD	22.5	9.8	4.2	40.9
Weak wash-in and persistent	all	28.6	13.7	2.4	80
	DSS	29.3	14.1	2.4	80
	DSD	23.0	8.6	5.4	45.3
Fast wash-in and plateau	all	3.7	3.8	0	26.6
	DSS	3.7	3.9	0	26.6
	DSD	3.9	2.8	0	10.9
Intermediate wash-in and plateau	all	9.7	4.4	0	26.2
	DSS	9.6	4.6	0	26.2
	DSD	10.1	3.0	3.7	16
Weak wash-in and plateau	all	5.0	3.9	0	44.3
	DSS	5.0	4.0	0	44.3
	DSD	4.4	2.5	1.6	12.4
Total tumor volume	all	9.7	29.1	0.1	313.1
	DSS	7.1	21.1	0.1	313.1
	DSD	29.9	60.4	0.5	249.7
Fast wash-in and wash-out	all	7.7	8.6	0	43.9
	DSS	7.6	8.8	0	43.9
	DSD	8.2	6.7	0	24.3
Intermediate wash-in and wash-out	all	12.1	9.6	0	55.7
	DSS	11.7	9.3	0	41.5
	DSD	16.0	11.2	2.5	55.7

Parameter	Subgroup	Mean	SD	Range	
Weak wash-in and wash-out	all	7.4	6.6	0	45
	DSS	7.2	6.4	0	41
	DSD	8.7	7.4	2	45

Cox regression

Covariate	b	SE	Wald	P	Exp(b)	95% CI of Exp(b)
Fast wash-in and persistent	0.02	0.02	1.38	0.24	1.02	0.9853 to 1.0609
Intermediate wash-in and persistent	-0.01	0.02	0.41	0.52	0.99	0.9578 to 1.0221
Weak wash-in and persis-tent	-0.04	0.01	7.57	0.006	0.96	0.9321 to 0.9883
Fast wash-in and plateau	0.01	0.04	0.11	0.74	1.01	0.9358 to 1.0982
Intermediate wash-in and plateau	0.03	0.04	0.62	0.43	1.03	0.9568 to 1.1086
Weak wash-in and plateau	-0.04	0.05	0.60	0.44	0.96	0.8613 to 1.0671
Total tumor volume	0.01	0.00	23.64	<0.0001	1.01	1.0066 to 1.0155
Fast wash-in and wash-out	0.01	0.02	0.28	0.60	1.01	0.9739 to 1.0468
Intermediate wash-in and wash-out	0.04	0.01	7.02	0.008	1.04	1.0103 to 1.0713
Weak wash-in and wash-out	0.03	0.02	1.46	0.23	1.03	0.9845 to 1.0681

Multivariate analysis: VAV

Covariate	b	SE	P	Hazard Ratio	95% CI
Time-to-peak enhancement (TTP)	0,70	0,16	<0,0001	2,02	1,47 to 2,78
Total tumor volume (TTV)	0,01	0,00	<0,0001	1,01	1,01 to 1,01
Persistent and weak wash-in	-0,06	0,02	0,0007	0,95	0,92 to 0,98

References

1. Edge, S., Byrd, D., Carducci, M. & Wittekind, C. *TNM Classification of Malignant Tumours*. (Springer, 2009).
2. Fattaneh, A. & Tavassoli, P. Tumours of the breast. in *World Health Organization Classification of Tumours. Pathology and Genetics.Tumours of the Breast and Female Genital Organs* 9–112 (IARC Press, 2003).
3. Elston, C. W. & Ellis, I. O. Pathological prognostic factors in breast cancer. I. The value of histological grade in breast cancer: experience from a large study with long-term follow-up. *Histopathology* **19**, 403–410 (1991).
4. Citation blinded for review process.
5. Wolff, A. C. *et al.* American Society of Clinical Oncology/College of American Pathologists guideline recommendations for human epidermal growth factor receptor 2 testing in breast cancer. *Arch. Pathol. Lab. Med.* **131**, 18–43 (2007).
6. Kreienberg, R., Kopp, I. & Lorenz, W. *Diagnostik, Therapie und Nachsorge des Mammakarzinoms der Frau S3 Leitlinie*. (Informationszentrum für Standards in der Onkologie (ISTO), 2004).
7. Deutsche Krebsgesellschaft e.V. (DKG), und Deutsche Gesellschaft für Gynäkologie und & Geburtshilfe (DGGG). *Interdisziplinäre S3-Leitlinie für die Diagnostik, Therapie und Nachsorge des Mammakarzinoms. Aktualisierung 2008*. (Zuckschwerdt Verlag, 2008).
